# Understanding the complexities of unexplained stillbirth in sub‐Saharan Africa: a mixed‐methods study

**DOI:** 10.1111/1471-0528.16629

**Published:** 2021-01-11

**Authors:** C Bedwell, K Blaikie, V Actis Danna, C Sutton, R Laisser, C Tembo Kasengele, S Wakasiaka, S Victor, T Lavender

**Affiliations:** ^1^ International Public Health Liverpool School of Tropical Medicine Liverpool UK; ^2^ School of Health Sciences University of Manchester Manchester UK; ^3^ Archbishop Antony Mayala School of Nursing Catholic University of Health and Allied Health Sciences Mwanza Tanzania; ^4^ Department of Public Health and Research Ministry of Health Headquarters Lusaka Zambia; ^5^ University of Nairobi Nairobi Kenya; ^6^ Perinatal Imaging and Health King's College London London UK

**Keywords:** Autopsy, communication, grief, mixed‐methods study, stillbirth, sub‐Saharan Africa

## Abstract

**Objective:**

To understand the complexities surrounding unexplained stillbirth for the development and implementation of culturally acceptable interventions to underpin care in Tanzania and Zambia.

**Design:**

Mixed‐methods study.

**Setting:**

Tertiary, secondary and primary care facilities in Mansa, Zambia, and Mwanza, Tanzania.

**Sample:**

Quantitative: 1997 women giving birth at two tertiary care facilities (one in each country). Qualitative: 48 women and 19 partners from tertiary, secondary and primary care facilities.

**Methods:**

Case review using data from a target of 2000 consecutive case records. Qualitative interviews with a purposive sample of women and partners, using a grounded theory approach.

**Results:**

A total of 261 stillbirths were recorded, with a rate of 16% in Tanzania and 10% in Zambia, which is higher than the previous estimates of 2.24 and 2.09%, respectively, for those countries. Women in both countries who reported a previous stillbirth were more likely to have stillbirth (RR 1.86, 95% CI 1.23–2.81). The cause of death was unexplained in 28% of cases. Qualitative findings indicated that not knowing what caused the baby to be stillborn prevented women from grieving. This was compounded by the poor communication skills of health professionals, who displayed little empathy and skill when counselling bereaved families.

**Conclusions:**

The stillbirth risk in both facilities was far higher than the risk recorded from national data, with women reporting a previous stillbirth being at higher risk. Women want to know the cause of stillbirth and an exploration of appropriate investigations in this setting is required. Providing health professionals with support and continuing training is key to improving the experiences of women and future care.

**Tweetable abstract:**

Stillbirths receive little investigation and are often unexplained. Communication with women about the death of their baby is limited.

## Tweetable abstract

Stillbirths receive little investigation and are often unexplained. Communication with women about the death of their baby is limited.

## Introduction

Stillbirth remains a major public health problem in low‐ and middle‐income countries (LMICs), with 98% of the 2.6 million estimated stillbirths occurring in these settings. Over half of stillbirths occur during labour and birth and are mostly preventable.^1^ Failure to prioritise stillbirth globally has meant that little has been done to reduce this burden, with many LMICs lacking the resources or political will to address the issue. Although the number of stillbirths has declined by 19.4% in the period 2000–2015, this represents an average annual rate of reduction (ARR) of 2%, which is less than both the maternal mortality (3%) and under‐five mortality rates (5.5%).^1^

The classification and reporting of stillbirth is limited in many LMICs, with differing definitions and inadequate reporting systems.^2^, ^3^ Stillbirths are under‐reported, particularly in rural areas where women may not attend facility‐based care.^4^ There is also considerable stigma associated with stillbirth in low‐income settings and the occurrence is often hidden from local communities,^5^ impacting the recording of deaths. Hence, the actual stillbirth rate is likely to be higher than the reported rate.

Women who suffer stillbirth may face health issues and require specialised care in future pregnancies. Although there is considerable evidence around the impact of stillbirth in high‐income settings, there is limited evidence from LMICs.^6^ Furthermore, the experiences and understanding of stillbirth from the perspective of partners in LMICs requires exploration, particularly given the stigma that women face in the community.

The slow reduction of stillbirths in LMICs makes it important to determine both the accuracy of numbers and the causes of death in order to tackle this problem. Few papers report on the causes of stillbirth in detail and difficulties in reviewing the cause of death have been reported.^7^ The availability of post‐mortem examination is extremely limited and, where available, the costs are often prohibitive to individuals.

The aim of this mixed‐methods study was to understand the complexities surrounding unexplained stillbirths to enable the development and implementation of interventions to support appropriate care for women in Tanzania and Zambia. In order to determine the extent and causes of stillbirth, we collected data from two countries with high stillbirth rates, Tanzania (22.4 per 1000 total births) and Zambia (20.9 per 1000 total births),^8^ as part of a larger programme of work investigating the prevention and management of stillbirth in sub‐Saharan Africa.

## Methods

A convergent parallel‐design mixed‐methods study was undertaken to enable a comprehensive understanding of the topic through the interpretation of different but complementary data. Quantitative data collection took place in a tertiary facility in Mansa region, Zambia, and Lake Zone, Tanzania, providing care for local women, women previously identified as high risk from pregnancy complications and women transferred from primary and secondary facilities with complications in labour. Qualitative data recruitment took place in antenatal and postnatal clinics in the tertiary facility of each region, along with primary and secondary facilities, as well as in the community. Primary facilities included small local clinics providing basic care for women at low risk. Secondary facilities provided a greater range of care during labour, but women deemed to be at high risk or with pregnancy complications required transfer to tertiary facilities. Recruitment and data collection for both aspects of the study were completed by research assistants, trained and mentored by UK and local research teams. The research assistants were all midwives (two in Zambia and three in Tanzania) with experience in recruitment and data collection.

### Quantitative data collection and analysis

To determine the extent and cause of stillbirth in the two regions, quantitative data were collected via a retrospective consecutive case note review, as part of a larger programme of work, between July and September 2018 at the main tertiary facility in each region. As this was a retrospective review, participant consent was not required, as confirmed by the ethics committees in each country. The target sample size of 1000 case notes from each country was chosen to enable high precision in the estimation of the risk of intrapartum stillbirth in each country, and to enable predictive modelling of factors related to stillbirth to be performed (results to be presented elsewhere). Data were collected from all women attending the participating facilities in the intrapartum period with a pregnancy of ≥28 weeks of gestation during the duration of the study. For the purposes of the research we adopted the definition of stillbirth used by the World Health Organization (WHO), as a baby born with no signs of life at or after 28 weeks of gestation.^1^ The case report form (CRF) was adapted from the WHO’s International Statistical Classification of Diseases and Related Health Problems – Perinatal Mortality (ICD‐PM) audit form.^9^ The form was reviewed and agreed by stakeholder groups (both in the UK and in the study countries) and the local Patient and Public Involvement (PPI) groups for applicability. The piloting of the CRF was undertaken in both study countries (*n* = 15) prior to data collection. Data were entered into a web‐based application, Research Electronic Data Capture (redcap),^10^ by trained research assistants in the study countries. Records were reviewed weekly online by the UK Research Associate (VAD) for missing data. Data validation of 10% of the total records was completed after data collection had ceased, demonstrating an error rate of less than 1% in both countries.

Data were anonymised and transferred into r 3.5.1 for analysis.^11^ Only data related to stillbirth are presented in this paper. Descriptive statistics were produced outlining how population characteristics differed by country. Characteristics of those with and without stillbirth allowed for comparison between groups. Data from pregnancies resulting in twins and neonatal death were excluded from this comparison.

### Qualitative data collection and analysis

Qualitative data were collected based on a Straussian grounded theory approach,^12^ which allows for the interpretation of complex social phenomena using an inductive and deductive approach.^12^, ^13^ The research assistants recruited and obtained informed consent from women and partners from tertiary and district facilities, local clinics and the community, representing both urban and rural areas. The participants in the qualitative sample differed from those in the quantitative sample. An initial purposive sample of three participants per group, in each country, were recruited: pregnant women, postnatal women with a live birth, postnatal women with a stillbirth, postnatal women with a near‐miss mortality, partners of pregnant women and partners of postnatal women. Participants were required to be 18 years of age and competent to consent. Theoretical sampling continued recruitment until data saturation was achieved.^14^ Semi‐structured interviews were conducted in the local language in a community setting, with demographic data collected to allow for the contextualisation of the findings. An interview guide and prompts enabled the researchers to explore key areas, whilst providing freedom to the participant to discuss areas of importance. This was adapted in line with developing theory. The translation and back‐translation of transcripts confirmed accuracy and ensured quality. Constant comparative analysis using the Strauss and Corbin approach was conducted by the UK and study country leads and the findings were confirmed by local PPI groups.^12^

### Funding

This research was funded by the National Institute for Health Research (NIHR) (16/137/53) using UK aid from the UK Government to support global health research. The views expressed in this publication are those of the author(s) and not necessarily those of the NIHR or the UK Department of Health and Social Care.

### Patient and public involvement (PPI)

The PPI groups were established in both countries with the aim of providing input into the study design and conduct, informed by cultural understanding.

## Results

### Quantitative

Data from 1997 records were analysed, following exclusions for three miscarriages misclassified as stillbirths. A total of 261 stillbirths (161 in Tanzania and 100 in Zambia) were recorded during the data collection period, of which 240 were singleton births (Table 1). The stillbirth rate was higher than anticipated: 16.1% in Tanzania and 10.0% in Zambia, compared with WHO estimates of 2.24 and 2.09%, respectively.^8^ Stillbirth occurred in 17.8% of twin births compared with 12.4% of singleton births.

**Table 1:** Birth outcomes

Pregnancy outcome indicators	Both countries			Tanzania			Zambia		
	Total	Singleton	Twin	Total	Singleton	Twin	Total	Singleton	Twin
Cases collected	1997	1938	59	1000	962	38	997	976	21
Total number of babies	2056	1938	118	1038	962	76	1018	976	42
Live birth (%)	1740 (85)	1645 (85)	95 (81)	844 (81)	784 (81)	60 (79)	896 (88)	861 (88)	35 (83)
Stillbirth (%)	261 (13)	240 (12.4)	21 (17.8)	161 (16)	146 (15)	15 (20)	100 (10)	94 (10)	6 (14)
Neonatal death (%)	49 (2)	48 (2)	1 (1)	32 (3)	31 (3)	1 (1)	17 (2)	17 (2)	0 (0)
Babies with unknown status (%)	6 (<1)	5 (<1)	1 (1)	1 (<1)	1 (<1)	0 (0)	5 (<1)	4 (<1)	1 (2)

The recorded causes of stillbirth are presented in Table 2; 28% of stillbirths (*n* = 73) were unexplained with no reason provided in the case notes (20.5% in Tanzania, *n* = 33; 40% in Zambia, *n* = 40). There was no record of a post‐mortem examination for all cases of stillbirth in both countries. Reasons were provided in some instances by attending clinicians, but it is unclear as to what extent examination of the stillborn baby occurred and to what extent these were subjective judgements. Therefore, where reasons were provided, it is uncertain as to whether these were causative factors or if they may have contributed to the stillbirth. It is possible that although an explanation was provided within the notes, these may not be completely accurate. Hence, although 72% (*n* = 188) of stillbirths appear to be explained, the attributed causes reported may be incorrect.

**Table 2:** Reported cause of stillbirth

Cause of stillbirth	Both countries (%)	Tanzania (%)	Zambia (%)
*n*	261	161	100
Fetal distress	53 (20.3)	35 (21.7)	18 (18.0)
Obstructed labour	32 (12.3)	24 (14.9)	8 (8.0)
Pre‐eclampsia/eclampsia	31 (11.9)	18 (11.2)	13 (13.0)
Antepartum haemorrhage	15 (5.7)	13 (8.1)	2 (2.0)
Cord prolapse/cord around neck	13 (5.0)	9 (5.6)	4 (4.0)
Anaemia	12 (4.6)	9 (5.6)	3 (3.0)
Other	8 (3.1)	4 (2.5)	4 (4.0)
Systemic Infection	7 (2.7)	5 (3.1)	2 (2.0)
Uterine rupture	7 (2.7)	3 (1.9)	4 (4.0)
Precipitate labour	4 (1.5)	4 (2.5)	0 (0.0)
Malaria	4 (1.5)	2 (1.2)	2 (2.0)
Prelabour rupture of membranes	1 (0.4)	1 (0.6)	0 (0.0)
Preterm labour	1 (0.4)	1 (0.6)	0 (0.0)
Unknown/unexplained	73 (28.0)	33 (20.5)	40 (40.0)

The characteristics of the 1885 participants who experienced singleton birth are presented in Table 3, following the exclusion of twins and neonatal death. The majority of participants were married (89%) and educated to primary school level (98%). Most women (78%) booked for care in the second trimester. Overall, 97.6% (*n* = 200) of participants experiencing stillbirth attended at least one antenatal clinic (ANC) visit, although there was a difference between the countries: 100% in Tanzania (*n* = 145); 91.7% in Zambia (*n* = 55). There are also more missing data from Zambia for both booking and antenatal attendance, however (Table 4). A smaller percentage of women experiencing stillbirth in this pregnancy attended for four or more visits (58.6% in Tanzania, *n* = 85; 45.0% in Zambia, *n* = 27). More participants experiencing stillbirth in the current pregnancy were transferred to a higher‐level facility during labour than those who did not experience stillbirth (46.2%, *n* = 111 versus 20.3%, *n* = 334), and were more likely to have an obstetrician present at birth (49.2%, *n* = 118 versus 30.6%, *n* = 504). More women in Zambia had experienced a previous stillbirth (65; 6.8% versus 18; 1.9%), with women who had experienced a previous stillbirth being more likely to experience a stillbirth in the current pregnancy (Tanzania, RR 2.17, 95% CI 1.11–4.24; Zambia, RR 2.19; 95% CI 1.29–3.71).

**Table 3:** Case note review of participant characteristics, by outcome (neonatal deaths excluded; singleton births only)

	Both countries (*n* = 1885)		Tanzania (*n* = 930)		Zambia (*n* = 955)	
	No stillbirth	Stillbirth	No stillbirth	Stillbirth	No stillbirth	Stillbirth
*n* (%)	1645 (87.3%)	240 (12.7%)	784 (84.3%)	146 (15.7%)	861 (90.2%)	94 (9.8%)
Mother’s age (years)						
Mean (SD)	27.3 (6.3)	27.6 (6.3)	28.1 (5.4)	28.5 (6.1)	26.6 (6.9)	26.2 (6.5)
18–35	1448/1645 (88.0%)	211/240 (87.9%)	707/784 (90.2%)	127/146 (87.0%)	741/861 (86.1%)	84/94 (89.4%)
<18	8/1645 (0.5%)	3/240 (1.2%)	7/784 (0.9%)	3/146 (2.1%)	1/861 (0.1%)	0/94 (0.0%)
>35	189/1645 (11.5%)	26/240 (10.8%)	70/784 (8.9%)	16/146 (11.0%)	119/861 (13.8%)	10/94 (10.6%)
Married						
No	172/1636 (10.5%)	33/240 (13.8%)	61/776 (7.9%)	24/146 (16.4%)	111/860 (12.9%)	9/94 (9.6%)
Yes	1464/1636 (89.5%)	207/240 (86.2%)	715/776 (92.1%)	122/146 (83.6%)	749/860 (87.1%)	85/94 (90.4%)
Unknown	9		8		1	
Education						
None or Primary	674/1373 (49.1%)	124/210 (59.0%)	366/775 (47.2%)	83/146 (56.8%)	308/598 (51.5%)	41/64 (64.1%)
Secondary	513/1373 (37.4%)	73/210 (34.8%)	298/775 (38.5%)	55/146 (37.7%)	215/598 (36.0%)	18/64 (28.1%)
Higher or vocational	186/1373 (13.5%)	13/210 (6.2%)	111/775 (14.3%)	8/146 (5.5%)	75/598 (12.5%)	5/64 (7.8%)
Unknown	302		9		293	
Gravida						
Median (IQR)	2 (1–4)	3 (1–4)	2 (1–4)	3 (1–4)	3 (1–5)	3 (1–5)
Parity						
Median (IQR)	1 (0–3)	2 (0–3)	1 (0–2)	2 (0–3)	1 (0–4)	2 (0–3)
History of stillbirth						
No	1575/1639 (96.1%)	221/240 (92.1%)	771/783 (98.5%)	140/146 (95.9%)	804/856 (93.9%)	81/94 (86.2%)
Yes	64/1639 (3.9%)	19/240 (7.9%)	12/783 (1.5%)	6/146 (4.1%)	52/856 (6.1%)	13/94 (13.8%)
Unknown	6		1		5	

**Table 4:** Attendance for care, by outcome (neonatal deaths excluded; singleton births only)

	Both countries (*n* = 1885)		Tanzania (*n* = 930)		Zambia (*n* = 955)	
	No stillbirth	Stillbirth	No stillbirth	Stillbirth	No stillbirth	Stillbirth
*n* (%)	1645 (87.3%)	240 (12.7%)	784 (84.3%)	146 (15.7%)	861 (90.2%)	94 (9.8%)
Gestation at booking						
1st tri.	212/1222 (17.3%)	38/198 (19.2%)	155/784 (19.8%)	28/145 (19.3%)	57/438 (13.0%)	10/53 (18.9%)
2nd tri.	956/1222 (78.2%)	147/198 (74.2%)	586/784 (74.7%)	106/145 (73.1%)	370/438 (84.5%)	41/53 (77.4%)
3rd tri.	54/1222 (4.4%)	13/198 (6.6%)	43/784 (5.5%)	11/145 (7.6%)	11/438 (2.5%)	2/53 (3.8%)
Unknown	465		1		464	
Any ANC visits						
No	3/1268 (0.2%)	5/205 (2.4%)	0/784 (0.0%)	0/145 (0.0%)	3/484 (0.6%)	5/60 (8.3%)
Yes	1265/1268 (99.8%)	200/205 (97.6%)	784/784 (100.0%)	145/145 (100.0%)	481/484 (99.4%)	55/60 (91.7%)
Unknown	412		1		411	
Number of ANC visits						
Mean (SD)	4.3 (1.2) [377 missing]	3.5 (1.2) [35 missing]	4.3 (1.2)	3.7 (1.0) [1 missing]	4.5 (1.2) [377 missing]	3.1 (1.6) [34 missing]
<4	289/1268 (22.8%)	93/205 (45.4%)	208/784 (26.5%)	60/145 (41.4%)	81/484 (16.7%)	33/60 (55.0%)
4+	979/1268 (77.2%)	112/205 (54.6%)	576/784 (73.5%)	85/145 (58.6%)	403/484 (83.3%)	27/60 (45.0%)
Unknown	412		1		411	
Distance from home to nearest health facility						
<30	1453/1604 (90.6%)	213/234 (91.0%)	699/745 (93.8%)	139/140 (99.3%)	754/859 (87.8%)	74/94 (78.7%)
31–60	126/1604 (7.9%)	13/234 (5.6%)	42/745 (5.6%)	0/140 (0.0%)	84/859 (9.8%)	13/94 (13.8%)
61–119	19/1604 (1.2%)	4/234 (1.7%)	3/745 (0.4%)	0/140 (0.0%)	16/859 (1.9%)	4/94 (4.3%)
120+	6/1604 (0.4%)	4/234 (1.7%)	1/745 (0.1%)	1/140 (0.7%)	5/859 (0.6%)	3/94 (3.2%)
Unknown	47		45		2	
Intrapartum transfer						
No	1308/1642 (79.7%)	129/240 (53.8%)	744/782 (95.1%)	90/146 (61.6%)	564/860 (65.6%)	39/94 (41.5%)
Yes	334/1642 (20.3%)	111/240 (46.2%)	38/782 (4.9%)	56/146 (38.4%)	296/860 (34.4%)	55/94 (58.5%)
Unknown	3		2		1	
Doctor or obstetrician available						
No	1141/1645 (69.4%)	122/240 (50.8%)	423/784 (54.0%)	65/146 (44.5%)	718/861 (83.4%)	57/94 (60.6%)
Yes	504/1645 (30.6%)	118/240 (49.2%)	361/784 (46.0%)	81/146 (55.5%)	143/861 (16.6%)	37/94 (39.4%)

For singleton stillbirths, the recorded time of death indicated that 42% (*n* = 101) occurred antenatally and 48% (*n* = 114) occurred in labour, with the timing of death unclear in 10% (*n* = 25) of cases. The condition of the fetus was noted as macerated in 45% (*n* = 109) of cases, fresh in 54% (*n* = 130) of cases and was not recorded in one case. This confirms our current understanding that the classification of time of death is difficult in LMICs,^15^ and that around half of deaths occur during labour.^7^, ^16^

### Qualitative

Forty‐eight interviews were conducted with women and 19 interviews were conducted with partners across the two countries. The demographics are provided in Table S1. The findings indicated that stillbirth was barely acknowledged by health workers and communication around stillbirth was poor. Failure to explain the reasons for stillbirth perpetuated elements of blame between women and health professionals.

#### ‘It just happens’

Data indicated that communication with women about the death of their baby was poor and frequently no explanation for the cause of death was given. The way in which the news was conveyed indicated that stillbirth was a routine occurrence and was afforded no value.

I just came to the hospital and they said, ‘it just happens’. (Woman, Tanzania)

In communicating with women, staff displayed an uncaring attitude and a lack of compassion, which may reflect the insignificance attributed to the event by health workers or may be a symptom of disrespectful care in general. Cultural belief may also play a part, whereby a stillborn baby is not viewed as human and, hence, is inconsequential. One partner recalled the behaviour of staff.

When she loses the baby, they don't even sympathise with the mother. They will say it is bad luck, go home, and that's all. (Partner, Tanzania)

The failure to acknowledge the stillbirth and its impact on the woman compounded the impression of the irrelevance of the loss to health professionals.

On discharge, no one talked or counselled me about my loss; to them, it was business as usual. (Woman, Zambia)

#### ‘Avoiding the question’

In many cases, women were not informed about the stillbirth at the time it occurred. The rationale for this was unclear but, in some situations, health workers appeared to be waiting for a relative to arrive and they would communicate the loss to them. The relative would then inform the woman. A partner described what the midwife said to him when he asked about the baby on arrival at the facility.

The child had died, we did not tell her mother; we were waiting for another person to come. (Partner, Tanzania)

On occasion, there appeared to be collusion between the staff and relatives over the loss, resulting in the concealment of the stillbirth from the woman.

When I woke up, I found two of my fellow women I was with sleeping next to their babies, I looked around I didn't see mine. When the nurse came in, I asked her where my baby was, she just said I will come back and left but she never came back. In the evening I insisted I wanted to know where my baby was, the nurse then told me that my baby was in intensive care unit for observation and left immediately, I wanted to ask what happened, but she left. It's like she was avoiding my question. In the night my grandmother brought me some porridge, I took this opportunity to ask her where my baby. She told me the same that my baby was in an intensive care unit for observation. The following day my grandmother came, she thought I was sleeping and then I overhead her telling my neighbour that I delivered a stillbirth, but told her not to tell me. (Woman, Zambia)

For many women, the failure to inform them in a timely manner meant that they had no opportunity to see their baby and relatives had often already buried the infant before the woman left hospital.

#### ‘I needed a proper answer’

When women were made aware of the stillbirth, they discussed wanting to understand the reasons for the death of their baby; however, they did not feel that they could ask the staff because of their attitude, bringing about a feeling of helplessness in the women.

I am even feeling frightened to ask the nurses they are too harsh … I am afraid I can't even ask, what am I going to ask, the child is already dead, even if I ask what will I do, the child is already dead? (Woman, Tanzania)

There appeared to be no will on the part of healthcare workers to help women and families understand the reasons for the stillbirth. The lack of provision of investigations, such as a post‐mortem examination, further limited parents’ understanding of the cause of the stillbirth.

Nobody knows what killed the baby up to now. We were just told the baby was dead, asked what killed the baby no [one] knew … I wish we could do post‐mortem but then, we do not have such services in our facilities. (Partner, Zambia)

In the absence of an explanation, women struggled to understand the pregnancy loss when they felt that they had done all they could to ensure a healthy pregnancy.

I have been attending all my clinic appointments. I made sure I ate well. I really don't know what happened. I needed a proper answer, but I did not get one … they just told me that my baby died in the uterus … but they didn't tell me what went wrong with my baby. (Woman, Tanzania)

#### ‘Blaming’

Blame was apparent, both for women and for health professionals. Health professionals would infer blame, indicating that the stillbirth was the fault of the woman.

I was in shock; it was unbelievable that I lost my baby just like that. The male nurse started blaming me for been lazy in pushing, I was so hurt but I could not speak. (Woman, Zambia)

Some women, having presented at the facility, blamed health professionals for the loss. Often, they felt that they were neglected or provided with poor care by the individuals caring for them.

The second baby, I gave birth at the hospital, but the nurses contributed to her death because they were not there to assist me when I needed them. (Woman, Tanzania)

## Discussion

### Main findings

This study highlighted a considerable difference between stillbirth estimates for countries and the actual stillbirth numbers recorded in the study health facilities. This may be accounted for in terms of the rigorous data collection of consecutive births in this study, combined with the transfer of women experiencing problems during labour into the facility. Furthermore, the areas from which the data were collected are in suburban and rural settings, which are estimated to have an increased stillbirth rate of up to 60% above national rates.^17^

The lack of availability of post‐mortem examination leaves an examination of the infant and clinical judgement as the only explanation of the death, which may be inaccurate. Furthermore, in almost a third of cases no attempt at explanation was provided in the case notes. Given that women who experience a stillbirth are more likely to experience recurrent stillbirth,^18^ this is concerning in respect of adaptation for future care. To improve clinical care and reduce stillbirth it is imperative that the cause is known to ensure appropriate care is instituted in future pregnancies.^19^ The higher risk of stillbirth amongst women with a previous stillbirth, as found in this study, reflects this and indicates a failure to understand and tackle individual health issues affecting pregnancy. Moreover, the role of health system issues that may impact on stillbirth, such as delays and transfer of care,^20^ remain an issue to be addressed. This lack of understanding compromises the aim of reducing global stillbirth rates, which is hampered by the failure to understand reasons for the deaths.^3^

### Strengths and limitations

This is one of the largest recent case reviews of stillbirth in Tanzania and Zambia; however, the review was reliant on the availability of information within the notes, with some data less likely to be recorded. This occurred particularly for Zambia and needs to be considered in interpreting the results. The mixed‐methods approach is a strength, allowing for a more complete understanding of the impact of unexplained stillbirth, and aligning these views with those of healthcare workers would provide an added dimension.

### Interpretation

The current position where women receive no offer of investigation to explain stillbirth is being challenged by the woman’s need for better understanding. A traditional post‐mortem examination, which includes invasive procedures, may not be acceptable in some cultures or to some parents.^19^ One aspect reported to be unacceptable is ‘cutting’ the infant.^21^, ^22^ There is growing evidence that alternatives, such as less‐invasive autopsy, are effective in determining the cause of death and may be more acceptable to women and their families.^19^ Non‐invasive and minimally invasive autopsies, which avoid dissection and instead use a combination of imaging, examination, biopsy and cultures, may be culturally more appropriate in these settings. Also, these are potentially less costly interventions, which is of relevance to LMICs.^23^ The identification of cause may lead to improvements in care, reducing the health, emotional and economic burden on women, families and society.^24^ Evidence suggests that women who receive and understand autopsy results are less likely to self‐blame and may feel some absolution for their antenatal actions,^25^ providing them with emotional closure.^22^, ^26^, ^27^ Health workers need to feel adequately trained to offer investigations. Less invasive techniques may be more acceptable to them in informing and consenting women to the process,^28^ as the cultural influences on women are also likely to affect health professionals’ views and understanding in the same setting. Given the poor understanding of stillbirth in this setting, autopsy is an area for consideration that requires further exploration, particularly with regard to the views of women, families, communities and health workers.

Poor communication about their stillbirth was a recurring feature for the women in our study. Women reported that health workers appeared to actively avoid the topic. The choice of language used was perceived by women to be dismissive and health workers’ attitudes prevented women from questioning them. The behaviour of staff is important to women and affects their experiences and ability to grieve following stillbirth.^29^ Health workers face systemic, emotional and knowledge‐based challenges in providing care, however, which may give the impression of a lack of concern for the women.^29^ Poor communication by health workers may stem from their own limited communication skills or discomfort in discussing death.^30^ Many health workers find discussing bereavement challenging and have expressed the need for further education to manage communication in such situations.^29^ The limited understanding and awareness of the causes of stillbirth may also impact their confidence in discussing the issue.^31^ That stillbirth is often unexplained may add to the health professionals’ feelings of inadequacy, and the lack of availability of investigations means that health workers are unable to offer women the potential for an explanation of the death. In some settings, a lack of support, and the fear of blame and litigation may lead health workers to avoid discussions.^24^ High numbers of stillbirth may lead to compassion fatigue,^32^ which may account for dismissive attitudes and an unwillingness to enter into discussions with distressed women and families.

Women were reluctant to raise questions about their stillbirth with health professionals, despite being keen to understand the cause. This may stem from the culture of blame experienced by women in sub‐Saharan Africa within their own communities.^5^ It may also relate to their gender and status, which often prevents them from having a voice in the community and health system. The failure to provide an explanation for the stillbirth adds to the blame that women experience, as there is no vindication of them or their perceived actions. That both health workers and some women seemed to accept stillbirth as a routine outcome is concerning and appears to confirm that there is limited will to change the accepted beliefs.

## Conclusion

Failure to identify the cause of death, coupled with a failure to communicate stillbirth appropriately, indicates that the current care for women experiencing stillbirth is not meeting their needs and may impact their future health and care. This work highlights two key research recommendations. First, although women perceive the existing process of communication as poor, further clarity is required to understand why this is the case. An additional exploration of the issues facing health workers in discussing stillbirth and the development of training interventions to overcome this is recommended. Second, the lack of real understanding of the cause of stillbirth requires more attention, not only to inform parents but also to ensure that women receive appropriate care in future pregnancies. Further work exploring the acceptability of autopsy, including different levels of investigation and an economic evaluation, is recommended. This is particularly important given the higher risk of stillbirth in each country amongst women who had previously experienced stillbirth.

In practice, it is recommended that health professionals need to be made aware of the impact of the language they use and their behaviour towards women, and the potential impact that their actions have on women and families.

Prior to this study, women have had little opportunity to voice their concerns or to have them heard. In addressing the issues identified, it is vital that women are the central focus to ensure that future care is developed appropriately to meet their needs.

### Disclosure of interests

None declared. Completed disclosure of interests forms are available to view online as supporting information.

### Contribution to authorship

All authors (CB, TL, RL, CK, SW, VAD, SV, KB and CS) contributed to the design of the study. CB, TL, RL, CK and SW analysed the qualitative data. KB, VAD and CS analysed the quantitative data. All authors (CB, TL, RL, CK, SW, VAD, SV, KB and CS) interpreted the data. CB drafted the first version of the article. All authors (CB, TL, RL, CK, SW, VAD, BV, KB and CS) commented on drafts of the article and have read and approved the final version for publication.

### Details of ethics approval

Ethical approval was obtained from the University of Manchester Ethics committee (ref. no. 2018‐4446‐6653, 18 July 2018), the National Health Research Authority and Ethics and Science Converge Institutional Review Board (ERES Converge IRB), Zambia (ref. no. 00005948, 29 June 2018) and the Catholic University of Health and Allied Sciences (CUHAS)/Bugando Medical Centre (BMC) Joint Ethical and Review Committee, Tanzania (ref. no. CREC/287/2018, 15 May 2018).

### Funding

This research was funded by the National Institute for Health Research (NIHR) (16/137/53) using UK aid from the UK Government to support global health research. The views expressed in this publication are those of the authors and not necessarily those of the NIHR or the UK Department of Health and Social Care.

### Acknowledgements

The authors would like to acknowledge the research assistants for their hard work and commitment to delivering this study; the PPI groups for their time and input in shaping the research; and the participants who freely gave up their time to share their views and experiences.

## Supporting information


**Table S1.** Participant demographics: interview study.

Supplementary Material

Supplementary Material

Supplementary Material

Supplementary Material

Supplementary Material

Supplementary Material

Supplementary Material

Supplementary Material

Supplementary Material

## Data Availability

Data available on request from authors.
