# Supplementary material for: Understanding the complexities of unexplained stillbirth in sub‐Saharan Africa: a mixed‐methods study
Source: BJOG. 2021 Jan 11;128(7):1206–14. doi: 10.1111/1471-0528.16629 (PMC8248405; doi:10.1111/1471-0528.16629)
Supplement: Supplementary file 1 — Table S1. Participant demographics: interview study. [file BJO-128-1206-s006.pdf]

Table S1. Participant demographics: interview study

	Tanzania		Zambia	
	**Women**	**Partner**	**Women**	**Partner**
	**N=21**	**N=10**	**N=27**	**N=9**
Age, median (range)	21 (18-41)	32 (26-50)	24 (18-45)	31 (25-56)
Marital status				
-Married	21	10	24	9
-Single	0	0	3	0
-Widowed	0	0	0	0
Education				
-Primary	5	1	9	3
-Secondary	15	6	15	4
-College	1	2	1	1
-Diploma	0	0	1	0
-Degree	0	1	1	1
-Other	0	0	0	0
Religion				
-Christian	21	10	27	8
-Muslim	0	0	0	1
Sampling group				
-Live birth	7	4	6	3
-Stillbirth	10	5	13	3
-Near miss	4	1	8	3
Employment				
-None/housewife	8	1	10	0
-Farmer	1	4	4	4
-Shop worker	3	1	5	1
-Tailor	3	0	4	0
-Clerical	4	0	3	1
-Business	1	4	1	3
-Nurse/midwife	1	0	0	0
